# Investigation of the Additional Use of a Single‑tufted Brush with a Standard Manual Toothbrush in Dental Students 

**DOI:** 10.3290/j.ohpd.c_2722

**Published:** 2026-06-19

**Authors:** Ayça Muhterem, Beyza Kaymaz, Şeymanur Genbay, Bilge Cansu Uzun Saylan, Pembe Keskinoğlu, Aliye Akcalı

**Affiliations:** a Ayça Muhterem Research Assistant and PhD Candidate, Dokuz Eylul University, Faculty of Dentistry, Department of Periodontology, Izmir, Turkey. Conceived and designed the study, collected data, drafted the manuscript, critically revised the manuscript, read and approved the final manuscript.; b Beyza Kaymaz Undergraduate Dental Student, Dokuz Eylul University, Faculty of Dentistry, Izmir, Turkey. Collected data, read and approved the final manuscript.; c Şeymanur Genbay Undergraduate Dental Student, Dokuz Eylul University, Faculty of Dentistry, Izmir, Turkey. Collected data, read and approved the final manuscript.; d Bilge Cansu Uzun Saylan Assistant Professor, Dokuz Eylul University, Faculty of Dentistry, Department of Periodontology, Izmir, Turkey. Critically revised the manuscript, read and approved the final manuscript.; e Pembe Keskinoğlu Professor, Dokuz Eylul University, Faculty of Medicine, Division of Basic Medical Sciences, Department of Biostatistics and Medical Informatics, Izmir, Turkey. Performed the statistical analysis, critically revised the manuscript, read and approved the final manuscript.; f Aliye Akcalı Professor, Dokuz Eylul University, Faculty of Dentistry, Department of Periodontology, Izmir, Turkey. Read and approved the final manuscript.

**Keywords:** dental plaque, dental plaque index, dental students, gingival index, toothbrushing.

## Abstract

**Purpose:**

Effective plaque control is essential for preventing and managing periodontal diseases. However, standard manual toothbrushing alone may be insufficient, especially in anatomically challenging areas. This study evaluated the adjunctive effect of a single-tufted brush on plaque and gingival indices when used with a standard manual toothbrush in periodontally healthy dental students.

**Materials and Methods:**

In this single-center, matched-pairs comparative study, 40 periodontally healthy dental students used a standard manual toothbrush alone on one side (control) of the mouth and a standard manual toothbrush plus a single-tufted brush on the contralateral side (experimental) for 2 weeks. Plaque and gingival indices were recorded at baseline and 2 weeks by a calibrated, blinded examiner. Data were analyzed using Friedman’s ANOVA for repeated measures and Dunn’s post-hoc test (α = 0.05).

**Results:**

Both groups showed statistically significant reductions in plaque and gingival indices over time (p < 0.001). Plaque index reduction did not differ statistically significantly between sides (p = 0.160), whereas gingival index reduction was statistically significantly greater on the experimental side (p < 0.001). Furthermore, 82.5% of participants expressed willingness to continue using the single-tufted brush.

**Conclusion:**

Adjunctive use of a single-tufted brush statistically significantly improved gingival health within a short period. It may be recommended as a practical supplementary oral hygiene aid, particularly for areas inadequately cleaned by standard manual toothbrushing alone.

The primary etiological factor in the initiation and progression of periodontal diseases is microbial dental plaque.^6,14^ Effective plaque control prevents the onset and progression of periodontal diseases, thereby also preventing tooth loss associated with these conditions.^8^

Toothbrushing is the most common and proven effective method used for mechanical plaque control. Under normal conditions, cleaning with a standard manual toothbrush alone does not ensure the effective removal of biofilm from all surfaces.^3^ The quality and quantity of plaque removal achieved through toothbrushing depend on the brushing technique, toothbrush design, frequency, and duration.^12^ Studies have shown that if oral hygiene procedures are not adequately maintained, gingivitis may develop within two weeks. Early signs of caries may appear if plaque accumulation persists for more than four weeks.^7,20^

Despite continuous design improvements in standard manual toothbrushes aimed at removing dental plaque, it has been reported that the average individual can remove only about 50% of dental plaque. A recent scoping review^17^ of randomized controlled trials confirmed that the plaque removal effectiveness of standard manual toothbrushes remains around 50% on average. Despite advancements in brushing techniques and toothbrush design, this rate has not significantly increased.^4,9^

Although toothbrushing is widely recognized as the most common and reliable method for mechanical plaque control, does not completely remove plaque, particularly from the proximal surfaces of the teeth and other anatomically challenging areas. For this reason, adjunct oral hygiene tools—including dental floss, interdental brushes, and tongue cleaners—are strongly recommended as complementary measures to toothbrushing. The combined use of these aids not only enhances the effectiveness of daily plaque removal but also plays a crucial role in maintaining optimal oral health by targeting regions that toothbrush bristles alone cannot adequately reach.^15,16^

Single-tufted brushes are also among the adjunct oral hygiene aids recommended in addition to the toothbrush. They have small brush heads with either a small cluster of tufts or a single tuft, and their handles can be straight or angled. The filaments are directed toward the area to be cleaned and applied using a rotational motion.^11^

The single‑tufted brush is designed for targeted cleaning of distal surfaces of posterior teeth and malpositioned or recessed teeth, as well as around fixed prostheses and orthodontic appliances, yet it appears to be less commonly used than dental floss or interdental brushes. Because the single‑tufted brush can be directed towards the gingival margin and intrasulcular area, it may offer advantages in removing biofilm where conventional brush heads have limited access.^11^ The present study aimed to compare the effects of a single‑tufted brush used in addition to a standard manual toothbrush with standard manual toothbrushing alone on plaque and gingival indices in contralateral regions of the same mouth in periodontally healthy dental students.

Despite widespread recommendation of adjunctive oral hygiene aids, the clinical evidence regarding the effectiveness of single‑tufted brushes remains limited and inconsistent. Most available studies have focused on specific patient groups, such as those undergoing orthodontic treatment or individuals with gingival recession, and there is a lack of controlled clinical data evaluating their adjunctive benefit in individuals with good baseline oral hygiene. In addition, the extent to which a single‑tufted brush provides additional benefit beyond standard manual toothbrushing, particularly in individuals with adequate oral hygiene habits, remains unclear. This randomized split‑mouth clinical trial therefore aimed to evaluate the adjunctive efficacy of a single‑tufted brush when used in conjunction with standard manual toothbrushing, on plaque and gingival indices in periodontally healthy dental students. The split‑mouth design allowed the standardization of inter‑individual variables such as manual dexterity, brushing habits, and motivation, thereby minimizing potential confounding factors and enhancing the internal validity of the findings.

## MATERIALS AND METHODS 

### Sample Selection

This study was designed as a randomized split-mouth clinical trial conducted at a single center. The research was conducted between November 2024 and March 2025, involving 40 volunteer participants selected from first, second, and third-year students of the Faculty of Dentistry at Dokuz Eylul University who met the inclusion criteria.

### Inclusion Criteria

Toothbrushing frequency at least twice a day 
≥18 years old
No systemic diseases
No regular medication use including anti-inflammatory or antibiotic medications within 3 months before or during the study
Non-smokers (defined as having no history of tobacco use within the last five years)

### Exclusion Criteria

Probing depth > 4 mm
Presence of periodontitis
Previous periodontal surgery
Presence of cervical or prosthetic restorations
Prior use of single-tufted toothbrushes
Undergoing orthodontic treatment or wearing orthodontic appliances

The study included periodontally healthy volunteers who met the following clinical criteria: a full natural dentition consisting of 28 teeth (excluding third molars), probing depths (PD) ≤3 mm at all sites, and no detectable interproximal clinical attachment loss (CAL = 0). The presence of cervical or prosthetic restorations was established as an exclusion criterion to ensure a standardized assessment of the single-tufted brush’s cleaning efficiency. This decision was based on the fact that the surface roughness (Ra values) of restorative materials differs importantly from that of natural tooth enamel. To eliminate potential variables arising from different surface characteristics and to evaluate the brush’s actual performance on a homogeneous surface, only natural cervical tooth surfaces were included in the study.

All procedures performed in this study involving human participants were conducted in accordance with the ethical standards of the institutional research committee and with the 1964 Declaration of Helsinki and its later amendments. The study was approved by the Non-Interventional Research Ethics Committee of Dokuz Eylul University Faculty of Medicine, Izmir, Turkey (Approval No: 2024/35-17, 23 October 2024). All participants were informed about the purpose and procedures of the study, and written informed consent was obtained prior to their participation. In addition, all participants provided consent for the use of their anonymized data for scientific research and publication purposes.

### Study Design and Participants

Before clinical evaluation, a 13-item questionnaire was administered to assess participants’ oral hygiene habits. Following this, participants were screened based on predefined inclusion and exclusion criteria, and eligible individuals were recruited for the study. Then, all participants were verbally educated about the modified Bass technique on a jaw model.^18,19^ In the control area, only a standard manual toothbrush was used (TePe Nova Soft, TePe Oral Hygiene Products; Malmö, Sweden), and in the experimental area, the standard manual toothbrush + a single-tufted brush (TePe Compact Tuft, TePe Oral Hygiene Products) were used. The sides of the participants’ mouths were randomly assigned using computer‑generated allocation. The use of the single-tufted brush was demonstrated with a rotational movement in the cervical areas, to be performed twice daily, and the application was explained in detail.

Plaque index (Silness and Löe, 1964) and gingival index (Silness and Löe, 1963) values were recorded at the start of the study and two weeks after the use of the single-tufted brush^11^ (Figs 1a and 1b). The index measurements following the use of the single-tufted brush were conducted two weeks after the initial measurement.^11^

**Fig 1a:**
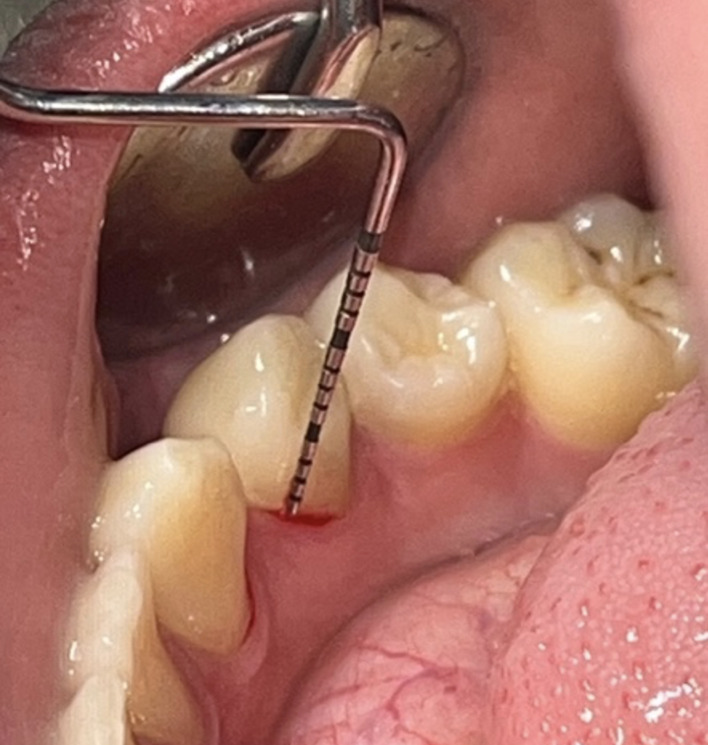
Gingival index measurement.

**Fig 1b:**
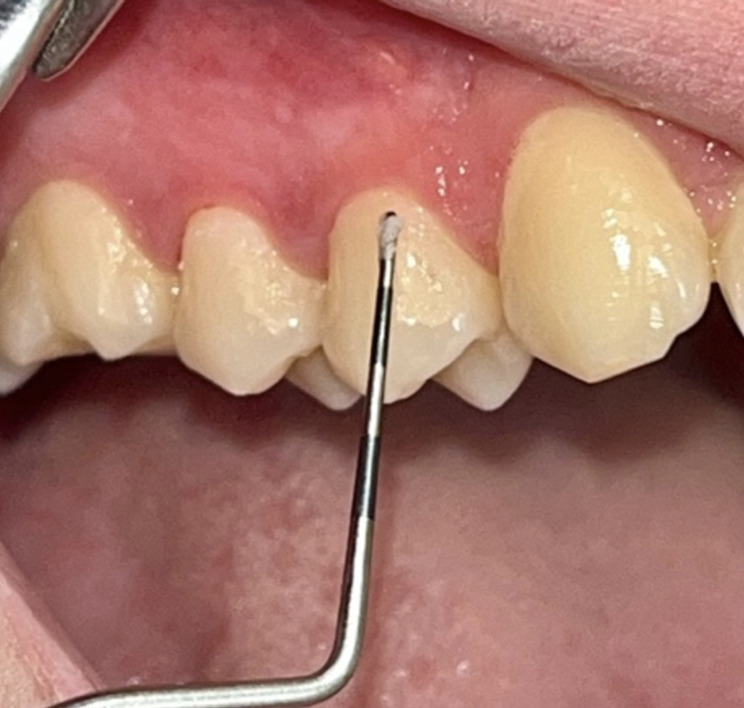
Plaque index measurement.

### Clinical Measurements

Clinical measurements were performed by a single calibrated examiner using a periodontal probe on six surfaces of each tooth (mesiobuccal, midbuccal, distobuccal, mesiolingual, midlingual, and distolingual) (κ = 0.91). Two weeks after the study began, the same person re-recorded plaque and gingival index values using the single-tufted brush. This study was designed as a randomized split-mouth clinical trial. The clinical examiner responsible for outcome measurements and the statistician performing the data analysis were blinded to the allocation of the single-tufted brush throughout the study period. Due to the nature of the intervention, participants could not be blinded to the use of the single-tufted brush. Thus, the study was examiner- and analyst-blinded but not participant-blinded.

Throughout the study, the personnel responsible for determining the quadrants (right or left) in which the single-tufted brush was to be used were different from the researcher who performed the periodontal index measurements. The researcher assessing the periodontal indices was unaware of both the allocation to the single-tufted brush intervention and the specific quadrant in which it was applied. All periodontal index measurements were conducted at baseline and after two weeks in the same dental unit, at the same time of day, and under standardized lighting conditions with identical light intensity.

Participants were given reminder cards to help them perform the applications regularly and were asked to mark their single-tufted brush usage twice daily (morning and evening) for two weeks. These cards were collected and checked one week after the start of the study. After the application, feedback was obtained from the students regarding the ease of use of the single-tufted brush and its contribution to oral hygiene.

### Statistical Analysis

Sample size calculation was performed for a paired comparison based on the primary outcome (gingival index). Assuming a medium effect size (d = 0.5), a power of 90%, and a statistical significance level of 5%, the minimum required sample size was calculated as 36 participants. To account for potential dropouts, 45 participants were initially planned. Ultimately, the study was completed with 40 participants who met the inclusion criteria.

The distribution of the data was assessed using appropriate normality tests. As the data did not meet the assumptions of normal distribution, non-parametric methods were applied. Continuous variables were presented as median (min–max), while categorical variables were expressed as frequencies and percentages (n, %).

In the split-mouth design, changes in plaque index and gingival index over time (baseline and 2 weeks) and between test (single-tufted brush) and control (standard manual toothbrush only) sites were analyzed using Friedman’s test for related samples. When statistically significant differences were detected, pairwise comparisons were performed using Dunn’s post-hoc test with appropriate adjustment for multiple comparisons.

The level of statistical significance was set at p < 0.05. All statistical analyses were performed using SPSS version 29.0 (IBM; Armonk, NY, USA).

## RESULTS

A total of 40 students from the Faculty of Dentistry, Dokuz Eylul University, voluntarily participated in the study. The median age of the participants was 20 years; 17 (42.5%) were female and 23 (57.5%) were male. Most of the students (77.5%) reported brushing their teeth at least twice daily, with 60% brushing for an average of 2 min. The majority (62.5%) used a medium-hard toothbrush, and 85% replaced their toothbrush every 3–6 months.

70% of the students reported interdental cleaning: 40% performed it 1–2 times per week, 15.4% performed it 3–4 times per week, and 17.5% more frequently. Moreover, 57.5% of the participants reported using a mouthrinse. With regard to single-tufted brushes, 70% of the students stated that they had not previously known about or used this type of brush.

**Fig 2:**
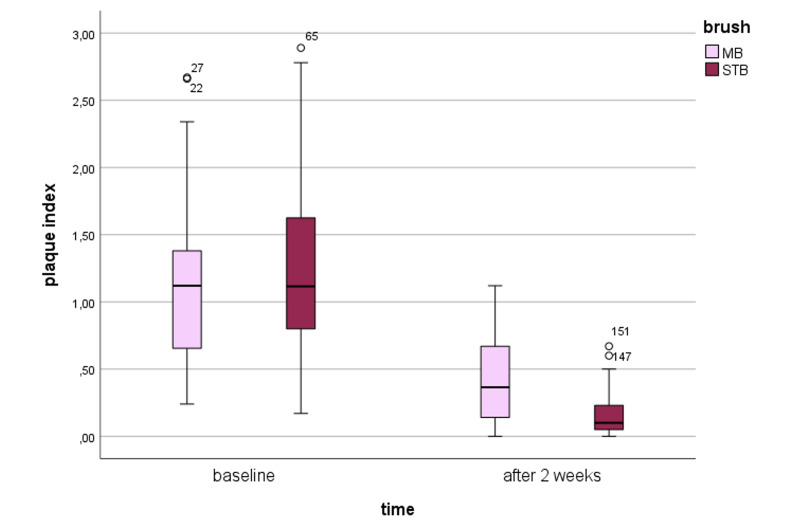
Box-and-whisker plots showing plaque index scores at baseline and after 2 weeks on the control side (standard manual toothbrush only, MB) and the experimental (single-tufted brush plus manual toothbrush, STB) side. No statistically significant between-side difference was observed at baseline, whereas both sides showed statistically significant within-side reductions over time (p < 0.001).

The demographic and oral hygiene characteristics of the participants are summarized in Table 1. For plaque index, no statistically significant baseline difference was found between the regions assigned to single‑tufted brush use (STB) and standard manual brush use (MB). After the two‑week intervention, both sides showed statistically significant within‑side reductions (p < 0.001), but the between‑side differences in plaque index remained statistically non‑significant (p = 0.160). Although baseline gingival index values did not differ statistically significantly between the groups, both sides exhibited marked reductions after two weeks, with a statistically significantly greater decrease observed in the STB region compared with the MB region (p < 0.001) (Table 2, Fig 2).

**Table 1:** Demographic characteristics and oral hygiene habits of the study participants


**Age (years) median/ (min–max)**	**20/ (18–21)**
**Sex (n/%)**	
Female	17/42.5
Male	23/57.5
**Year of study**	
1st	13/32.5
2nd	15/37.5
3rd	12/30.0
**Toothbrushing frequency (per day)**	
Once	1/2.5
Twice	31/77.5
Three times or more	8/20.0
**Toothbrushing duration (minutes)**	
1	1/2.5
2	24/60.0
3	10/25.0
≥4	5/12.5
**Toothbrush replacement frequency**	
<3 months	4/10.0
3–6 months	34/85.0
>6 months	2/5.0
**Use of interdental cleaning aids**	
Yes	28/70.0
No	12/30.0
**Frequency of interdental cleaning (per week)**	
None	11/27.5
1–2 times	16/40.0
3–4 times	6/15.0
≥5 times	7/17.5
**Knowledge and use of single-tufted brush**	
Aware and used	2/5.0
Aware but not used	10/25.0
Not aware and not used	28/70.0
Use of mouthrinse	
Yes	23/57.5
No	17/42.5
**Self-rated oral hygiene (0–10 scale)**	
5	1/2.5
6	6/15.0
7	19/47.5
8	9/22.5
9	5/12.5
**Type of standard toothbrush**	
Soft	14/35.0
Medium	25/62.5
Hard	1/2.5


**Table 2:** Time-dependent comparison of plaque index between the experimental side (single-tufted brush + standard manual toothbrush) of the dental arch and the control (standard manual toothbrush used alone) side

Plaque index	Median	(Min–Max)	p
STB0	1.1	(0.2–2.9)	Total p < 0.001* STB0-MB0 p = 1.000*** STB0-STB1 vs MB0-MB1 < 0.001** STB1-MB1 p = 0.160****
MB0	1.12	(0.2–2.7)	
STB1	0.1	(0.0–0.7)	
MB1	0.4	(0.0–1.1)	
STB0: single-tufted brush, baseline; STB1: single-tufted brush, after two weeks; MB0: standard manual brush, baseline; MB1: standard manual brush, after two weeks. *Friedman’s Variance Analysis – total P plaque; **post-hoc Dunn’s test p: STB0-STB1, MB0-MB1; ***post-hoc Dunn’s test p: 0.160; ****post-hoc-p: STB1-MB1.			

**Fig 3:**
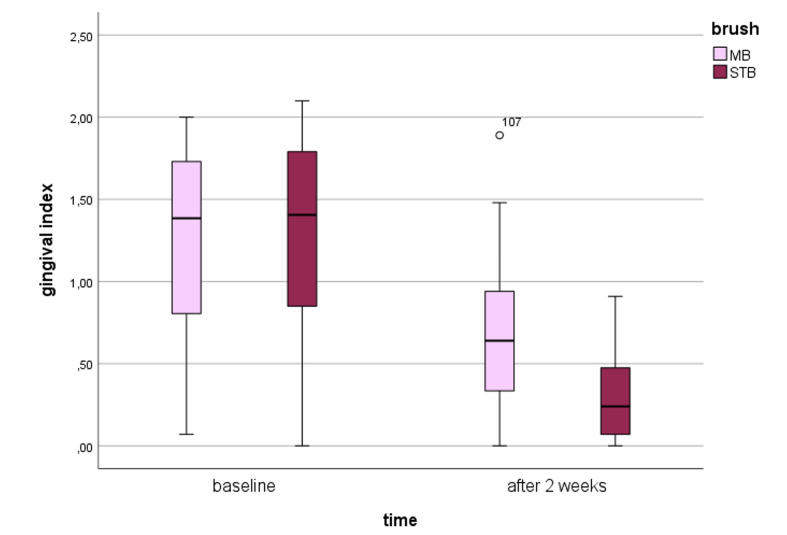
Box-and-whisker plots showing gingival index scores at baseline and after 2 weeks on the control (manual toothbrush only, MB) side and the experimental (single-tufted brush plus manual toothbrush, STB) side. Both sides showed statistically significant within-side reductions, with a statistically significantly greater decrease on the STB side at 2 weeks (p < 0.001).

Although no statistically significant difference was observed in baseline gingival index values between the groups (STB0 median: 1.4, min: 0.0 – max: 2.1; MB0 median: 1.4, min: 0.1 – max: 2.0), a marked reduction in gingival index was recorded in both groups after the two-week intervention. The side on which students used the single-tufted brush in addition to standard manual toothbrushing showed a greater decrease in gingival index compared to the side brushed solely with the standard manual brush (STB1 median: 0.2, min: 0.0 – max: 0.9; MB1 median: 0.6, min: 0.0 – max: 1.9). This difference was statistically significant (p < 0.001) (Table 3, Fig 3).

**Table 3:** Time-dependent comparison of gingival index between the experimental side (single-tufted brush + standard manual toothbrush) of the dental arch and the control (standard manual toothbrush used alone) side

Gingival index	Median	(Min–Max)	p
STB0	1.4	(0.0–2.1)	Total p < 0.001 t STB0-MB0 p = 1.000 tt STB0-STB1/ MB0-MB1/ STB1-MB1 < 0.001ttt
MB0	1.4	(0.1–2.0)	
STB1	0.2	(0.0–0.9)	
MB1	0.6	(0.0–1.9)	
t: Friedman’s ANOVA – bleeding (p); tt: post-hoc Dunn’s test – p, all pairwise comparisons except baseline; ttt: post-hoc Dunn’s test – p: baseline, single-tufted vs standard manual brush.			

### Student Feedback

According to the survey conducted after the application, 82.5% of the students stated that they wanted to continue using the single-tufted brush, while 90% expressed that the use of this brush in oral care is necessary. The satisfaction level was mostly concentrated in the mid-to-high range. When asked to rate their satisfaction with the use of the single-tufted brush on a scale of 1 to 10, 35% gave a score of “8,” 15% gave “9,” and 20% gave “10,” with a total of 70% scoring between 8 and 10. Regarding the difficulty of use, 67.5% of the participants rated it between “0–6” out of 10 (Table 4).

**Table 4:** Student feedback

Will you continue using the single-tufted brush?	(n/%)
Yes	33/82.5
No	7/17.5
Please rate your satisfaction with the use of the single-tufted brush (0 – not satisfied at all, 10 – very satisfied).	
3 4 5 6 7 8 9 10	1/2.5 2/5.0 12/30.0 2/5.0 4/10.0 14/35.0 6/15.0 8/20.0
Please rate the difficulty of using the single-tufted brush on a scale of 0 to 10 (0 – not difficult at all, 10 – very difficult).	
1 2 3 4 5 6 7 8 9 10	2/5.0 4/10.0 7/17.5 7/17.5 6/15.0 7/17.5 1/2.5 2/5.0 3/7.5 1/2.5
Do you think the single-tufted brush should be used in oral care?	
Yes	36/90.0
No	4/10.0


## DISCUSSION

This study represents the first investigation in the literature to evaluate the effectiveness of a single-tufted brush, used in addition to a standard manual toothbrush, on one half-side of the dentition in individuals with comparable levels of manual dexterity. Data collected regarding participants’ toothbrushing habits, frequency of single-tufted brush use, and toothbrush replacement practices demonstrated a higher level of oral hygiene awareness compared to the general population. This characteristic provided a suitable model for examining the additional benefits of the single-tufted brush in individuals who already maintain good oral hygiene. Since the patient’s hand coordination and motor skills are essential during oral hygiene, the single-tufted brush was evaluated on different arch regions (right and left) within the same student. Participants’ opinions regarding the ease of use of the single-tufted brush and its contribution to oral hygiene were also assessed after its use.

Although 70% of participants reported performing interdental cleaning and 77.5% reported brushing twice daily, 70% indicated that they were unfamiliar with and had never previously used a single-tufted brush. This finding suggests that the single-tufted brush remains relatively unknown, even among individuals with high oral health awareness.

In our study, a statistically significant reduction in gingival index scores was observed in the regions where the single-tufted brush was additionally used for a two-week period. This clinical improvement, recorded within a short timeframe, suggests that the use of a single-tufted brush may facilitate plaque control in localized areas that are often less accessible to conventional manual toothbrushes. The specific design of the single-tufted brush, which allows better access to difficult-to-reach sites, may be a potential factor contributing to the observed decrease in gingival inflammation indicators.

Interestingly, these gingival improvements were not accompanied by a statistically significant between‑side difference in plaque index, suggesting that even modest local changes in plaque accumulation or composition, or improved access to specific sites, may translate into clinically detectable changes in gingival inflammation.

With regard to plaque index findings, reductions were observed in both the single-tufted brush and control regions. This result highlights the positive impact of the toothbrushing instructions provided at baseline and the follow-up process on participants’ oral hygiene.

In a clinical study evaluating the plaque-removing efficacy of single-tufted vs standard manual toothbrushes, the percentage reduction in plaque scores achieved with single-tufted brushes was found to be statistically significantly higher than that of manual brushes in the maxillary buccal interproximal, marginal, and mandibular lingual interproximal regions. This finding indicates that single-tufted brushes may serve as an effective tool for plaque control, particularly in the posterior teeth.^10^

In a study conducted by Cunha et al,^5^ the effectiveness of a single-tufted toothbrush, used alone or in combination with a manual toothbrush, was evaluated for plaque control in periodontally healthy orthodontic patients. The combination of the single-tufted and manual toothbrushes was found to be effective in plaque removal in these patients.^5^

Makeeva et al^13^ investigated the effect of single-tufted toothbrush use in patients with Class I gingival recession. The group using a standard manual toothbrush in combination with a single-tufted brush showed statistically significantly lower plaque index values at 1 week and 1 month compared to the group using only a manual toothbrush (p < 0.001). In patients with Class I gingival recession, the use of a standard manual toothbrush together with a single-tufted brush was shown to contribute to oral hygiene and achieve more effective cleaning in the cervical regions.

Aykol-Şahin et al^2^ compared the effectiveness of three types of toothbrushes—manual, orthodontic, and single-tufted—over a three-month follow-up period in 36 patients with gingivitis using fixed appliances. By conducting a long-term evaluation in patients with fixed appliances, which are highly prone to plaque retention, the study demonstrated the effectiveness of the single-tufted toothbrush in reducing gingival inflammation.^2^ On the other hand, in a different study involving 26 patients with fixed lingual orthodontic appliances, the plaque-removing efficacy of orthodontic and triple-headed manual toothbrushes, used alone or in combination with a single-tufted toothbrush, was evaluated. The study found that the use of a single-tufted brush in addition to a standard manual toothbrush did not provide any additional benefit in patients with fixed lingual appliances.^1^

Considering these few studies, although the plaque-removing efficacy of single-tufted brushes has not been conclusively established, the combined use of a standard manual toothbrush with a single-tufted brush is recommended for certain patient groups, such as those undergoing orthodontic treatment or with gingival recession. Standard manual toothbrushes may be insufficient for plaque removal in specific areas of the dentition.

Given that patient manual dexterity and motor skills play an important role in the use of oral hygiene tools, the present study aimed to determine the effects of single-tufted and standard manual toothbrushes on plaque and gingival indices when applied to different parts of the dental arches (right vs left) within the same individual. Comparing different regions within the same subject effectively controls for inter-individual variability (e.g., hand coordination, personal motivation, salivary characteristics), thereby increasing the reliability of the results. The researcher who performed the index measurements was unaware of which sides were assigned as the experimental or control sides, which contributed to the methodological robustness of the study.

In addition, the provision of toothbrushing instruction (modified Bass technique) and regular follow-ups resulted in a statistically significant reduction in both plaque and gingival indices. These findings may indicate that patient education and motivation alone can contribute to improvements in oral hygiene.

This study has several limitations. First, the single‑tufted brush was used as an add‑on to existing oral hygiene, which inevitably increased the total brushing time on the experimental side of the dentition and may partly explain the observed improvements in clinical indices. Second, the follow‑up period was limited to 2 weeks, which does not allow conclusions about long‑term maintenance. Furthermore, the inclusion of dental students with relatively high oral hygiene awareness and motivation limits the generalizability of the findings to the broader population. Finally, the Hawthorne effect may have increased participants’ motivation and adherence, potentially exaggerating the impact of oral hygiene instruction and the intervention. Nevertheless, due to the split‑mouth design and short follow‑up period, these results should be interpreted as indicative of a potential adjunctive benefit rather than definitive evidence of a causal effect of the single‑tufted brush itself.

Tooth surfaces do not present a uniform geometry due to variations in arch curvature and tooth inclination. Therefore, the clinical use of conventional manual toothbrushes at a constant 45-degree angle across all areas of the oral cavity is unlikely in practice. During actual brushing, variation in angulation from tooth to tooth and from surface to surface is expected, with this variability being more pronounced at canines, molars, and lingual surfaces. In this study, the inclusion of the single-tufted toothbrush was intended to evaluate its potential to improve manual cleaning efficiency in areas where conventional brushes have limited adaptability, despite the anticipated variability in brushing angle. The small and focused brush head of the single-tufted toothbrush is designed to facilitate access and local control even when deviations from the ideal brushing angle occur.

The inclusion of a single-tufted brush in the daily oral hygiene routine, in addition to the use of a manual toothbrush, may contribute to the reduction of gingival inflammation even within a short period. However, patients should be instructed on the proper brushing technique, and oral hygiene motivation should be maintained through regular follow-ups.

Future research could include studies that control total brushing time to be equivalent in the control group. Long-term studies with larger, more heterogeneous populations exhibiting varying levels of oral hygiene are also warranted.

## CONCLUSION

The adjunctive use of a single‑tufted brush in combination with a standard manual toothbrush was associated with a greater reduction in gingival index scores over a 2‑week period compared with standard manual toothbrushing alone. The split‑mouth design, by controlling for inter‑individual factors such as manual dexterity, enabled a more standardized evaluation of the local effects of the intervention. These findings suggest that a single‑tufted brush may offer additional short‑term benefits in oral hygiene practices, particularly for cleaning cervical and hard‑to‑reach areas in motivated individuals with good baseline oral hygiene. However, further long‑term studies involving more diverse populations are needed to confirm these observations and to determine their clinical relevance in routine practice.

## ACKNOWLEDGEMENTS

The authors would like to thank all the students who volunteered to participate in this study. This study was supported by the TÜBİTAK 2209 Research Project Support Program for Undergraduate/Graduate Students.

## References

[ref1] Ashkenazi M, Salem NF, Garon S, Levin L (2015). Evaluation of orthodontic and triple-headed toothbrushes when used alone or in conjunction with single-tufted toothbrush in patients with fixed lingual orthodontic appliances: A randomized clinical trial. N Y State Dent J.

[ref2] Aykol-Sahin G, Ay-Kocabas B, Mert B, Usta H (2024). Effectiveness of different types of toothbrushes on periodontal health in orthodontic patients with gingivitis: A randomized controlled study. BMC Oral Health.

[ref3] Bergenholtz A, Brithon J (1980). Plaque removal by dental floss or toothpicks: An intra-individual comparative study. J Clin Periodontol.

[ref4] Chakrapani S, Polepalle T, Kolaparthy L, Kuntcham R, Adurty C, Sirigadha S (2014). An evaluation of plaque removal efficacy of five commercially available toothbrushes: A comparative clinical study. Int J Dent Sci Res.

[ref5] Cunha LDD, Peruzzo DC, Costa LA, Pereira ALP, Benatti BB (2018). Effect of a single-tufted toothbrush on the control of dental biofilm in orthodontic patients: A randomized clinical trial. Int J Dent Hyg.

[ref8] Kwon T, Lamster IB, Levin L (2021). Current concepts in the management of periodontitis. Int Dent J.

[ref10] Lee DW, Moon IS (2011). The plaque-removing efficacy of a single-tufted brush on the lingual and buccal surfaces of the molars. J Periodontal Implant Sci.

[ref16] Seemann R, Kison A, Bizhang M, Zimmer S (2001). Effectiveness of mechanical tongue cleaning on oral levels of volatile sulfur compounds. J Am Dent Assoc.

[ref17] Slot DE, Wiggelinkhuizen L, Rosema NA, Van der Weijden GA (2012). The efficacy of manual toothbrushes following a brushing exercise: a systematic review. Int J Dent Hyg.

[ref19] Weng L, Wen J, Cui G, Liang J, Pang L, Lin H (2023). Comparison of modified bass, rolling, and current toothbrushing techniques for the efficacy of plaque control: A randomized trial. J Dent.

[ref20] Zhang M, Lan J, Zhang T, Sun W, Liu P, Wang Z (2021). Oral health and caries/gingivitis-associated factors of adolescents aged 12–15 in Shandong province, China: A cross-sectional oral health survey. BMC Oral Health.

